# Overview of the Maturation Machinery of the H-Cluster of [FeFe]-Hydrogenases with a Focus on HydF

**DOI:** 10.3390/ijms19103118

**Published:** 2018-10-11

**Authors:** Marco Bortolus, Paola Costantini, Davide Doni, Donatella Carbonera

**Affiliations:** 1 Department of Chemical Sciences, University of Padova, Via F. Marzolo 1, 35131 Padova, Italy; donatella.carbonera@unipd.it; 2 Department of Biology, University of Padova, Viale G. Colombo 3, 35131 Padova, Italy; davide.doni.2@phd.unipd.it

**Keywords:** hydrogenases, [FeFe]-hydrogenases, HydF, HydG, HydE, maturases

## Abstract

Hydrogen production in nature is performed by hydrogenases. Among them, [FeFe]-hydrogenases have a peculiar active site, named H-cluster, that is made of two parts, synthesized in different pathways. The cubane sub-cluster requires the normal iron-sulfur cluster maturation machinery. The [2Fe] sub-cluster instead requires a dedicated set of maturase proteins, HydE, HydF, and HydG that work to assemble the cluster and deliver it to the apo-hydrogenase. In particular, the delivery is performed by HydF. In this review, we will perform an overview of the latest knowledge on the maturation machinery of the H-cluster, focusing in particular on HydF.

## 1. Introduction

**Figure 1:**
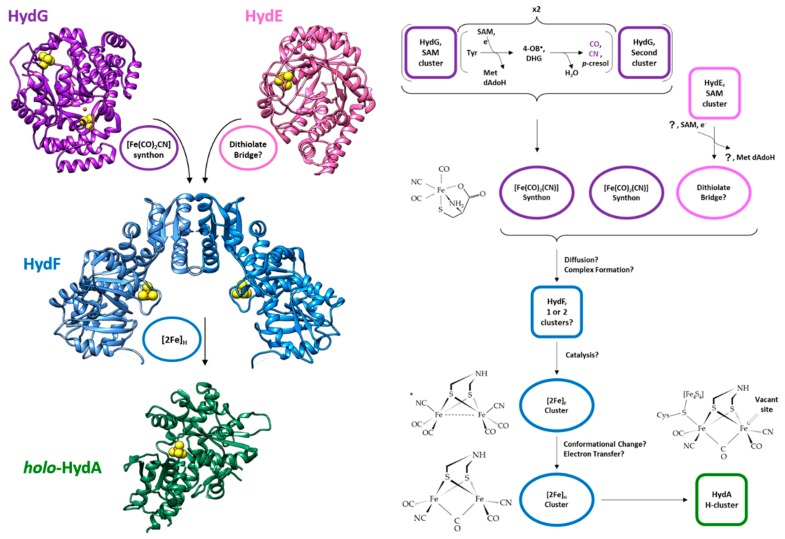
Scheme of the maturation process of the [2Fe]_H_ sub-cluster of [FeFe] hydrogenase. On the left side, we show the structures and active sites of the enzymes involved in the maturation: purple, HydG from *T. italicus* (pdb.id 4WCX) with the first active site, the SAM cluster on top, and the second active site on the bottom; pink, HydE from *T. maritima* (pdb.id 3CIW); blue, HydF) from *T. melaniensis* (pdb.id 5KH0); green, HydA, from *C. reinhardtii* (pdb.id 3LX4), without the complete active site. On the right side, a more detailed scheme of the reactions involved in the process. The enzymes are indicated as squares (the color scheme is conserved); the products of the enzymes are shown as ovals of the same color of the enzymes producing them. Some relevant chemical structures are reported, from top to bottom: the synthon; the [2Fe]_F_ cluster; the [2Fe]_H_ cluster; and the active site of the holo-HydA with the full H-cluster. The question mark denotes unknown substrates/products; the asterisk denotes the attachment point of the [2Fe]_F_ sub cluster to HydF. For the acronyms, please refer to the abbreviation section.

Hydrogen metabolism is one of the most ancient processes of life, and today it is at the center of growing attention in the field of bioenergy (or bio-inspired) production technologies [1,2,3,4,5]. Hydrogenases are the enzymes responsible for the biological production or consumption of hydrogen in all domains, Archaea, Bacteria, and Eukarya [6,7]. They are divided into three classes based on their metal cofactors [7,8]: [Fe]-hydrogenases [9]; [NiFe]-hydrogenases [10]; [FeFe]-hydrogenases [11]; [NiFeSe]-hydrogenases [12]. [Fe]-hydrogenases, found only in methanogenic Archaea, are the smallest group and are restricted to a single function, they catalyze reversible reaction of methenyltetrahydromethanopterin with H_2_ to methylenetetrahydromethanopterin and H^+^. [NiFe]-hydrogenases are widespread among all bacteria families, including archaea, eubacteria and cyanobacteria. [FeFe]-hydrogenases have been found in some unicellular green algae, such as *Chlamydomonas reinhardtii*, as well as in strict anaerobes, fungi and protists. For the most part, hydrogenases likely function either in recycling reduced electron carriers that accumulate during anaerobic fermentation through proton reduction or in coupling H_2_ oxidation to energy yielding processes. Indeed, both [NiFe]- and [FeFe]-hydrogenases catalyze the reversible conversion of protons into hydrogen 2H^+^ +2e^−^ ⇄ H_2_ in conditions of strict anaerobiosis; [FeFe]-hydrogenases are usually involved in the forward reaction, while [NiFe]-hydrogenases in the backward reaction, although there are a number of exceptions [13,14]. Both classes of enzymes are oxygen sensitive, and hydrogen production is strongly inhibited by aerobic conditions, whereas [FeFe]-hydrogenases are irreversibly inactivated during catalysis by trace amounts of O_2_, in most cases [NiFe]-hydrogenases react reversibly with O_2_, giving rise to a mixture of inactivated states [15,16,17,18]. This sensitivity to molecular oxygen is one of the most critical drawbacks that have seriously limited the use of recombinant hydrogenases as biotechnological tools so far, and several recent studies aim at overcoming this problem from different angles [2,19,20,21]. In fact, growing interest in exploiting particularly robust and more O_2_-tolerant enzymes has become a major driving force for understanding the hydrogenases biogenesis and catalytic mechanisms, in vivo and in vitro. An additional problem for potential technical applications of biomimetic catalysis aimed at H_2_ production by artificial hydrogenases is linked to the synthesis of their active site in a functional form [22,23]: recent advances have shown that in vitro activation of the recombinant enzymes using synthetic precursors is feasible [24]. It is worth noting that among all hydrogenases, [FeFe]-hydrogenases (HydA) show an unsurpassed H_2_ release activity of up to 8.000 μmol H_2_ min^−1^ mg^−1^ [25]: this efficiency is based on the unique and peculiar design of the cofactor responsible for their catalytic activity, the so-called H-cluster, which requires a dedicated protein machinery to be assembled [26]. As discussed in detail below, to date this maturation pathway is still incompletely characterized. Figure 1 shows that this site is a complex organometallic center composed of two halves, a standard cubane (called [4Fe-4S]_H_) anchored to the protein backbone via four cysteine residues, one of which acts as the linker for the other half, a complex cluster (called [2Fe]_H_) in which two Fe atoms are coordinated each by a CO and a CN^−^ and bridged by a third CO and an aza-dithiolate moiety, the nature of which had been long debated [27,28]. As soon as structural and functional details of the [FeFe]-hydrogenases active site were known, production of bioinspired catalysts for hydrogen production became a major goal in the field and this prompted the molecular studies of the H-cluster assembly. As for other FeS-proteins, the maturation process allowing the assembly of the H-cluster follows some basic common biosynthetic rules (reviewed in [29]), and can be divided in two main coordinated steps, i.e., the synthesis of a cluster precursor on a scaffold protein and the accurate delivery of this precursors to the target apoprotein, which culminates with its assembly into the polypeptide chain. The biosynthesis of the H-cluster is further complicated by the existence of the unusual non-protein ligands, CO, CN^−^ and dithiolate, and by its composite double iron, unique in nature. It is not surprising that, whereas several systems for the biogenesis of more conventional [4Fe-4S] and [2Fe-2S] clusters have been thoroughly characterized in bacteria as well as in different eukaryotic intracellular compartments [29], despite crucial advancements made in recent years, important gaps remain in the understanding of the molecular pathway leading to the assembly of the [FeFe]-hydrogenase H-cluster, and eventually allowing the enzyme activation. Three highly conserved proteins, at first found in *C. reinhardtii* and then in all microorganisms containing a [FeFe]-hydrogenase [30], have been shown to form the minimal synthetic machinery for the assembly of this site: HydE, HydG and HydF. Based on several in vitro experimental evidences, it has been proposed that the whole HydE/HydF/HydG maturation machinery would be dedicated to the synthesis and insertion of the [2Fe] subcluster, with its ligands, into a hydrogenase containing a [4Fe-4S] unit that would be performed by a housekeeping FeS clusters biogenesis system [31]. In fact, the division of the H-cluster in two sub-clusters is also reflected by their different synthetic paths: [4Fe-4S]_H_ is assembled and inserted into the hydrogenase by the general iron-sulfur clusters assembly machinery in a first step, while the [2Fe]_H_ is assembled by the three specific maturase proteins, as outlined in Figure 1 [30,32,33]. All three maturases are essential for the maturation of HydA, as shown by the lack of hydrogen production in the absence of one of the three proteins [30,34]. HydF is a homodimeric GTPase belonging to the family of the P-loop NTPases [35] and containing a FeS cluster binding consensus sequence [30], and is the core of the H-cluster biogenesis pathway, since it plays a dual role in the maturation process acting as scaffold upon which the final form of the [2Fe]_H_ cluster is assembled and as carrier to insert it into the hydrogenase [36,37]. HydG and HydE, both monomeric radical-SAM (*S*-adenosyl-l-methionine) proteins, synthesize different parts of the H-cluster and interact with HydF to assemble it. While many details of these maturation proteins have been elucidated in recent years, the exact sequence of events and intermediates of the maturation process is still unknown. 

The principal methods that have been used to characterize hydrogenases and their active sites are crystallography, fourier-transform infrared spectroscopy (FT-IR), electron paramagnetic resonance spectroscopy (EPR). Crystallography beside providing the molecular structures of the enzymes, also helped in the identification of the molecular mechanisms through co-crystallization experiments with putative substrates. However, most of the information on the mechanisms of action of the hydrogenases and on their maturation process, has been obtained through spectroscopic methods, mainly EPR methodologies and FT-IR, often coupled together. Conventional EPR spectroscopy (i.e., continuous-wave EPR) provides a wealth of information on FeS clusters of the enzymes: different kinds of FeS clusters have different EPR spectra. Additionally, since EPR is only sensitive to paramagnetic states, it can help to define the oxidation state of the metal centers in different conditions. When coupled to isotopic labeling or mutagenesis experiments, pulsed EPR methods allow to identify the residues bound to the metal centers of the active site in the different functional states through the detection of the electron-nuclei hyperfine coupling. Finally, EPR has been coupled to the site-directed spin labeling method (SDSL) to study the changes in structure and dynamics of the enzymes in solution. FT-IR resulted to be unique in its ability to differentiate both the nature of the H-cluster ligands, and their mode of binding. By a careful analysis of the molecular stretching modes, the CO and CN^−^ ligands and their orientation have been defined. In addition, the stretching modes sensitive to the iron oxidation state have allowed to identify the EPR-silent cluster states. 

In this work, we will review the updated knowledge on the maturation machinery of the H-cluster, highlighting the questions that are still open, focusing in particular on the structure and catalytic mechanisms of HydF. A first section will be devoted to the individual enzymes and a second one to the discussion of the overall process.

## 2. The maturases

### 2.1. HydE

HydE is the least characterized of the three maturation enzymes, while its structure has been solved and HydE has been assigned to the radical SAM superfamily of enzymes, its substrates and products are still uncertain. While the exact role of HydE in the maturation process is not well-defined, it has been suggested to provide the bridging di (thiomethyl) amine ligand of the H cluster [38]. 

**Figure 2:**
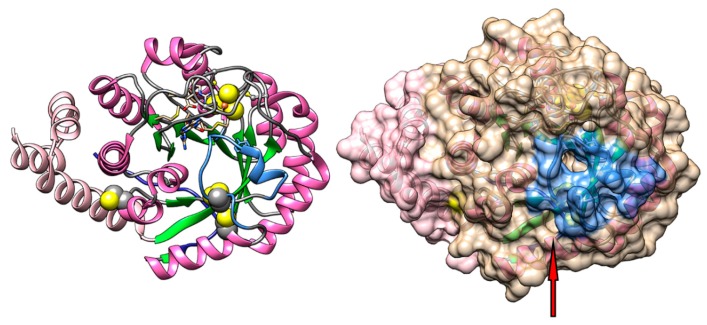
HydE (pdb.id 3CIW) from *T. maritima.* Left, cartoon representation of the protein, showing the protein from the top of the internal cavity. In magenta (helix) and green (strand) the TIM-barrel fold; in pale pink the n-terminal helices; in dark blue (on the other side of the protein) the c-terminal loop; in light blue the putative “lid” region governing the access to the active site; the [4Fe-4S] cluster forming the active site is in sphere representation; and the cysteine residues not binding the cluster are also in sphere representation. Right, same view as before but with the surface shown: the accesses to the internal cavity are in the center of the light blue zone and close to the two cysteines as shown by the red arrow.

The structure of HydE has been solved only for the enzyme from *Thermotoga maritima* (pdb.id 3CIW) [39], see Figure 2. HydE from *T. maritima* adopts a distorted triose-phosphate isomerase (TIM)-barrel fold, where 8 α-helices and 8 parallel β-strands (shown in dark pink and green, respectively, in Figure 2) alternate along the peptide backbone, these last forming a large internal cavity where a molecule of SAM and additional substrates can be accommodated. Relative to the standard TIM-barrel fold, HydE has additional helices at the n-terminus and an additional strand at the c-terminus (shown in pale pink and dark blue, respectively, in Figure 2). The protein hosts one [4Fe-4S] cluster in the n-terminal part which is highly conserved and has been identified as the active site; this cluster faces the internal cavity of the enzyme, and it is coordinated by a SAM molecule in the crystal structure. A secondary [FeS] cluster binding site is present, about 2 nm away from the SAM cluster, but this secondary site does not seem to be essential for the maturation process, the cysteines of the secondary site are shown as spheres in Figure 2. The stoichiometry of the secondary cluster is not exactly defined in the crystal structures, varying from [2Fe-2S] to [4Fe-4S] [40], and EPR spectroscopy data have shown that full cluster reconstitution leads to two [4Fe-4S] clusters in the enzyme [38].

Based on the crystal structure, the access to the internal cavity has been hypothesized to be regulated by a region of the protein directly above the active site, a loop-helix-loop structure acting as a “lid” (shown in light blue in Figure 2) with a strictly conserved aromatic motif, Y*XX*Y. The protein surface on this region shows one main access point in the “lid” region and a second one that is open in the absence of the secondary cluster (indicated by the red arrow in Figure 2). Both accesses are narrow, but, combined with dynamic conformational fluctuations of the protein in solution, could allow substrates and products to diffuse to the active site. A third access to the internal cavity, closed in the static protein structure, rests on the other side of the cavity relative to the active site, covered by the c-terminal loop. 

While it is certain that the dithiolate bridge comes from HydE, the exact substrates and products of HydE are unknown. HydE belongs, like HydG, to the radical SAM superfamily of enzymes, which perform a homolytic cleavage of the C5′–Sδ bond of SAM at a [4Fe-4S] cluster, to yield a reactive 5′-deoxyadenosyl radical species (5′-dA•), used to usually extract a hydrogen atom from a substrate, thus generating a highly reactive carbon centered radical which is used in further synthetic steps. At variance with the general mechanism, it has been shown that HydE can react directly on a sulfur atom to form a C-S bond without passing through the hydrogen abstraction [41]. However, without knowledge of the actual substrate, it is unclear if this unusual reaction is part of the physiological mechanism involved in the maturation process. Nevertheless, it has been suggested that the substrate of HydE contains a thiol functional group [38]. Additionally, the SAM chemistry is performed on the main cluster, but the role of the secondary, less conserved, cluster in the function of the enzyme is still unknown.

### 2.2. HydG

HydG has been well-characterized, its crystal structure has been solved for different organisms and its function has been recently established in detail. HydG, belonging as HydE to the radical-SAM superfamily, is a bifunctional enzyme performing the synthesis of a Fe-containing complex with CO and CN^−^ ligands that acts as a synthon of the [2Fe]_H_ cluster.

**Figure 3:**
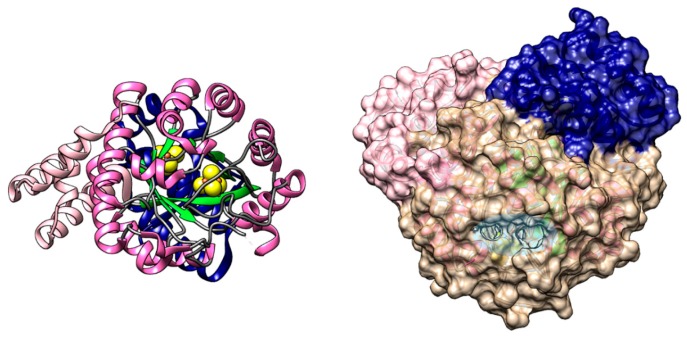
HydG (pdb.id 4WCX) from *T. italicus.* Left, cartoon representation of the protein, in the figure we show the protein from the top of the internal cavity. In magenta (helix) and green (strand) the TIM-barrel fold; in pale pink the N-terminal helices; in dark blue (on the other side of the protein) the C-terminal domain; and the [4Fe-4S] and [5Fe-4S] clusters forming the two active sites at the opposite sides of the cavity are in sphere representation. Right, a rotated view of the protein with the surface shown: the access to the internal cavity is in the center shaded in light blue.

The structure of HydG has been solved for two organisms, *Carboxydothermus hydrogenoformans* (pdb.id 4RTB) [42] and *Thermoanaerobacter italicus* (pdb.id 4WCX) [43], the latter shown in Figure 3. HydG, similarly to HydE, has a distorted TIM-barrel fold, 8 α-helices and 8 β-strands (shown in dark pink and green, respectively, in Figure 3), with additional helical domains at the n-terminus and c-terminus (about 80 amino acids long, shown in pale pink and dark blue, respectively, in Figure 3). It has a large internal cavity at the edge of which two distinct active sites, both iron-sulfur clusters, are present. The first is a [4Fe-4S] cluster with the conserved structural motif of a radical-SAM active site, and it is located at the top of the cavity of the TIM-barrel fold. The second site is a peculiar [4Fe-4S] cluster bridged by a μ_2_ sulfide ion (or a non-proteic Cys) to a labile dangling Fe atom, and it is located at the bottom of the TIM-barrel cavity about 2.4 nm away from the first cluster, bound to the additional helical domain at the C-terminus. The second cluster is the site at which a Fe(CO)_2_CN synthon is assembled (see the next paragraph). The internal cavity of HydG is connected to the protein surface by a channel through which the products and substrates are thought to diffuse, shaded in light blue in Figure 3. The opening/closing of the channel located at the top of the cavity, close to the SAM active site, is thought to be governed by a loop region with a conserved Arg residue that partakes in Tyr binding, and is analogous to *Streptomyces actuosus* tryptophan lyase [42,43].

HydG has long been recognized to provide the CO and CN^−^ ligands of the [2Fe]_H_ cluster. Recently, mostly through a combination of isotopic labeling and pulsed EPR experiments the role of HydG in the maturation pathway has been further expanded [41,44,45,46,47]: it has been shown to synthesize the synthon of the [2Fe]_H_ cluster, a low-spin iron complex, Fe(CO)_2_(CN)Cys. The assembly of the synthon is a multi-step process involving both active sites of the enzyme. The first active site utilizes the radical-SAM mechanism similarly to HydE (see above) to cleave a tyrosine into *p-*cresol, one molecule of CO, and one of CN^−^ via a dehydroglycine intermediate. *p*-cresol migrates out of the protein cavity following the opening of the active site, while the other products migrate to the bottom of the cavity at the second active site. The second active site assembles the synthon: the dangling Fe atom, initially coordinated by a free cysteine, water, and histidine from the backbone, is coordinated by two CO and one CN^−^ produced at the first active site while the second CN^−^ cleaves the formed synthon from the [4Fe-4S] cluster. Note that in the proposed mechanism the stoichiometry of the overall formation of the synthon involves the conversion of two molecules of tyrosine at the first site to produce all the necessary ligands.

### 2.3. HydF

HydF holds a central role in the maturation pathway, but while its homodimeric structure has been crystallized, and its dual role, as a scaffold upon which the [2Fe]_H_ cluster is assembled and carrier of the cluster into the apo-HydA, is universally recognized, a detailed knowledge of its mechanisms of action is still missing.

**Figure 4:**
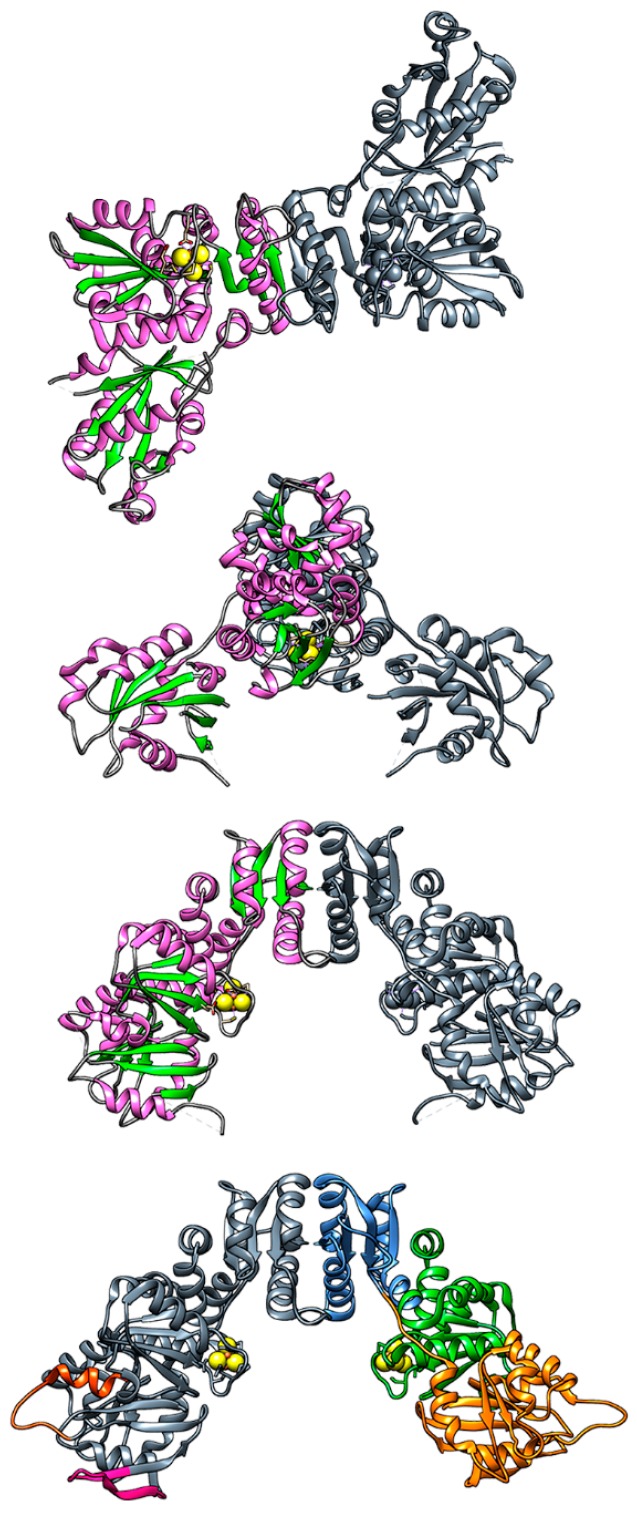
Top, HydF (pdb.id 5KH0) from *T. melaniensis* viewed from three angles (bottom of the cavity, and two views of the sides rotated 90° one relative to the other). To highlight the homodimeric assembly of the protein only one monomer has been colored according to secondary structure: in magenta the α-helices, in green the β-strands, the [4Fe-4S] cluster is in sphere representation. Bottom, reconstruction of the whole protein structure in the absence of GTP based on HydF from *T. melaniensis* (see text) including the missing amino-acid stretches. The left monomer has the reconstructed stretches colored in purple (switch 1 region) and dark orange (switch 2 region). The right monomer has been colored to highlight the three protein domains: GTPase domain in orange, dimerization domain in blue, and cluster-binding domain in green.

The structure of a recombinant HydF from *Thermotoga neapolitana* (pdb.id 3QQ5) has been solved without the [4Fe-4S] cluster [48], which is known to be part of the holo-protein as derived by EPR of the protein in solution-state [37,49,50,51,52,53,54,55], and more recently complete with the cluster from *Thermosipho melaniensis* (pdb.id 5KH0, used for Figure 4) and from *T. maritima* (pdb.id 5LAD) [56]. HydF is a homodimeric protein with three domains per monomer. The N-terminus holds the GTPase domain, the central domain is the dimerization domain, and the c-terminus holds the [4Fe-4S] cluster-binding domain. While HydE and HydG are globular, the homodimeric structure of HydF adopts an open fold with a skewed inverted V shape, with the two GTPase domains located at the outermost part of the dimer and twisting away from the plane formed by the other two domains (as shown in Figure 4). This fold creates a large open “cavity” just below the dimerization interface with the active [4Fe-4S] cluster site of each monomer in its center. The cluster is solvent-exposed as befits an active site that needs to interact with several different proteins, as shown in Figure 4. 

#### 2.3.1. GTPase Domain

The GTPase domain at the N-terminus (orange in Figure 4), is the least resolved in the crystal structures, it has high B factors, and is not stabilized in the crystal by direct contact with other molecules [56]. Its fold is similar to that of other GTPases: six β-strands, five parallel and one anti-parallel, assemble into a large sheet, with three α-helices flanking this sheet on one side and two α-helices on the other. Recently, through sequence and structure analysis, it has been shown that the GTPase domain has a high homology to analogous domains of other small K^+^-dependent GTPases, and as such it should act as a molecular switch triggering a conformational change [57]. Two regions have been identified as switch regions: switch 1 (residues 31–46, located directly above the nucleotide binding site) not solved in any crystal structure, and switch 2 (residues 68–86), solved only for HydF from *T. neapolitana*. Switch 1 is the most conserved in function and sequence among different K^+^-dependent GTPases. Upon GTP binding, the switch 1 region of K^+^-dependent GTPases exhibits a change from a β-sheet to a structured loop region with specific interactions with K^+^ and Mg^2+^ ions mediated by conserved Asn residues [58]. This structural change is highly conserved, and likely adopted by HydF as well. On the other hand, the switch 2 region, which in HydF comprises a loop ending in an α-helix, has a high variability among the proteins belonging to different species and thus the structure adopted by HydF in the presence of GTP is not easily predicted starting from that resolved in the absence of GTP. Based on these considerations, we here present a model of the whole protein in the absence of GTP constructed with the UCSF Chimera package [59]. The model is based on the structure of HydF from *T. melaniensis*, pdb.id 5KH0, on top of which the two switch regions have been modeled. The switch 2 region was taken from the structure from HydF from *T. neapolitana* (pdb.id 3QQ5) where it is resolved. The switch 1 region was taken from the GDP-bound soluble N-terminal domain of FeoB from *Streptococcus thermophilus* (pdb.id 3LX8), a membrane protein that imports Fe^2+^ [60], taken as the reference structure of a K^+^-activated GTPase. The two reconstructed regions are highlighted in Figure 4 (bottom): switch 1 (residues 31–46) in purple and switch 2 (residues 68–86) in dark orange.

#### 2.3.2. Dimerization Domain

The second domain in the sequence is responsible for the formation of the HydF dimer (blue in Figure 4). An extended stretch of residues, about thirteen amino-acids long, connects the dimerization to the GTPase domain. The domain is composed of four parallel β-strands and three α-helices. The four-stranded parallel β-sheets of each monomer are coupled in an antiparallel way to form a continuous eight-stranded β-sheet. Additional stabilization comes from the interactions between the neighboring α-helices and the loop regions, to a degree that depends on the species. Note that, so far, it is not clear why HydF needs to be dimeric to partake in cluster maturation, since a single [4Fe-4S] cluster is potentially sufficient to anchor the [2Fe]_H_ cluster. On the other hand, a close look at the HydF structure indicates that the total buried surface due to the dimerization is large enough to support the existence of a stable physiological dimer, which assumes a sort of left-handed helical shape leaving both the putative FeS cluster and GTP-binding sites exposed to the solvent and giving a large protein surface for contacts with possible partners, such as the other two maturases and/or the apo-[FeFe]-hydrogenase. Thus, the dimer could be essential to provide the central open cavity to bind alternatively HydE, HydG, or the domain of HydA containing the H-cluster. This issue will be discussed in greater detail below.

#### 2.3.3. Cluster Binding Domain

The c-terminal domain hosts the putative enzyme active site, where the [4Fe-4S] cluster is predicted to act as an anchor for the [2Fe]_H_ assembly and delivery (green in Figure 4). The domain is composed of four β-strands and five α-helices arranged in a complex way to bring the three highly conserved Cys residues belonging to the highly conserved iron-sulfur cluster-binding motif (CxHx46-53HCxxC) spatially close [48,56]. Site-specific mutagenesis analysis coupled to EPR spectroscopy have shown that while cysteine residues are essential for the cluster assembly of HydF, the conserved histidines are not, and do not belong to the cluster coordination sphere [52]. However, the histidines are essential for the [2Fe]_H_ cluster assembly, possibly partaking in this process via hydrogen bonding to the synthons. The crystal structure of HydF from *T. melaniensis* revealed that the fourth ligand of the cluster in the absence of the synthon comes from a highly conserved acidic residue (Glu in this organism) coordinating via the carboxylate [56]. HYSCORE spectroscopy studies had already shown that the fourth ligand is an oxygen in a variety of organisms and that this ligand is easily exchangeable, as expected since the fourth position is the one where the [2Fe]_H_ cluster is anchored [52,54,55]; to date, the only known exception coming from the cluster of *Clostridium acetobutylicum*, where a nitrogen was found, however the possibility that the His-tag at the N-terminus of the recombinant protein, which is located spatially close to the cluster, interferes with the natural coordination must be taken into account. The putative binding pocket of the [2Fe]_H_ cluster is located in a cleft at the interface between the second and third domains and is characterized by a positively charged surface. The loop that holds the fourth ligand likely changes conformation upon de-coordination of the acidic residue for the binding of the synthon, a model has been proposed comparing the two existing crystal structures, with and without the cluster [56].

In addition to the [4Fe-4S] cluster, there is evidence from EPR spectroscopy that as-isolated and chemically reconstituted HydF can also host a [2Fe-2S] cluster [61,62,63,64,65], which is otherwise lacking in all solved HydF crystal structures [48,56]. Given this discrepancy, it is still unclear whether the presence of a [2Fe-2S] cluster represents a physiological state, a state of partial cluster maturation, or simply arises from an incomplete reconstitution due to the in vitro conditions. Since there are no other binding motifs in HydF, and its relaxation properties suggest that the [2Fe-2S] cluster is located at least 2.5 nm away from the main cluster, it is likely bound in the same binding site in a different monomer, with a mixed occupancy of the sites in the two monomers, or a subset of proteins binding only a single type of cluster [62]. Finally, while there is a consensus about the role of the [4Fe-4S] cluster as the anchor for the [2Fe]_H_ cluster, the role of [2Fe-2S] cluster is completely undefined.

The role of HydF as a scaffold and carrier in the maturation process is widely accepted based on both structural and functional studies [24,36,48,66], but several unclear points on its function are still present.

#### 2.3.4. Tetrameric Form

HydF adopts in the crystal structures and in the purification process a tetrameric form (or more precisely forms a dimer of dimers), less abundant than the native dimeric form. The quaternary structure of the tetramer is uncertain since in the three available crystal structures the tetrameric assembly differs greatly and it is likely influenced by the crystal packing [56]. It is possible to separate the two forms in the recombinant protein via chromatographic methods, but the dimer and tetramer are in dynamic equilibrium [64]. A regulatory role has been attributed to the tetrameric form of HydF, acting as a switched-off state of the enzyme, since the tetrameric fractions are much less active in assembling the cluster [62]. 

#### 2.3.5. [2Fe]_H_ Cluster Precursor

In a recent work [65], the precursor to the [2Fe]_H_ cluster present in HydF (called [2Fe]_F_ cluster) has been suggested to be structurally different from the one present in HydA, based on FT-IR data (see Figure 1 for its structure). [2Fe]_F_ has not been detected by EPR spectroscopy suggesting it is diamagnetic. The [2Fe]_F_ cluster form is coordinatively saturated and bridged to the [4Fe-4S] cluster of HydF by a cyanide ligand. Relative to the [2Fe]_H_ cluster, the [2Fe]_F_ cluster would lack the bridging CO molecule and the relative orientation of the CO and CN- ligands relative to the dithiolate bridge is different, see Figure 1. Another difference that has been highlighted is the different redox state of the sub clusters, which would imply that the [4Fe-4S] cluster of HydF might be redox active and involved in the change of the redox state going from [2Fe]_F_ to [2Fe]_H_, a step that might play a role in the transfer of the cluster to HydA.

#### 2.3.6. Role of the GTPase Domain

**Figure 5:**
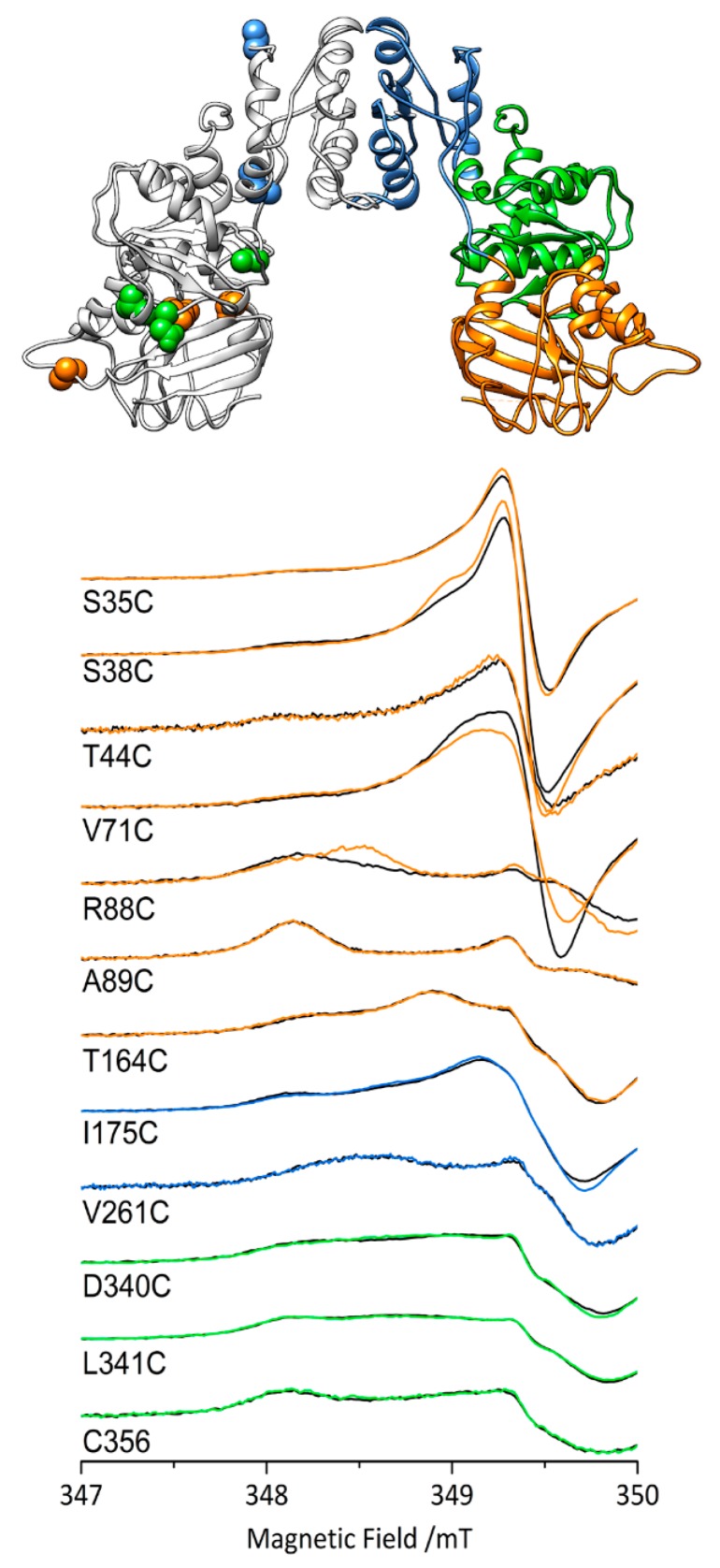
Top. The structure of HydF from *T. neapolitana* (pdb.id 3QQ5) with the mutated labeling sites highlighted in the same color of the domain they are located in (orange, GTPase; blue, dimerization; green, cluster binding). Bottom. EPR spectra (zoom of the low field region) of HydF from *T. neapolitana* spin labelled at the indicated positions: black, the spectra in the absence of GTP; colored lines, the spectra in the presence of GTP. The figure is based on the results reported in reference [57].

Site-specific mutagenesis analysis revealed that the HydF GTPase consensus motifs are essential for the [FeFe]-hydrogenase maturation and activation [34,66]. NTPases are commonly involved in the assembly of metal cofactors of FeS proteins [67], mediating either the metal delivery to the active site or the transfer of the whole cluster to the target apoprotein. Experimental evidences excluded a role of HydF GTPase activity in the transfer of H-cluster precursor to the [FeFe]-hydrogenase [68], and an involvement of the GTPase domain in the interaction with the two other maturases has been suggested [66,68]. As reported above, the HydF crystal structures showed that this domain includes a flexible loop region which could rearrange upon GTP binding, thus facilitating the interaction with the maturation partners (see next paragraph). The changes in structure and backbone dynamics in the presence of GTP were investigated by EPR spectroscopy coupled to site-directed spin labeling (SDSL) and CD spectroscopy both in the full enzyme [57] and in the isolated domain [69]. EPR spectra in the presence of non-hydrolysable analogues and transition state mimics of GTP showed that GTP binding, and not its hydrolysis, triggers the switch. The binding of GTP causes a change in backbone dynamics diffused throughout the whole protein (Figure 5): the largest changes are in the GTPase domain (S38, V71, R88), at the interface between the GTPase and cluster-binding domain (D340), or between the GTPase and dimerization domains (I175), while they are nonexistent in the core of dimerization domain proper (V261). All buried residues that were labeled do not change spectra upon GTP addition, suggesting that they do not get exposed irrespective of the domain (A89, T164, L341). DEER (Double Electron-Electron Resonance) experiments, measuring the distances between couples of spin labels, carried on double mutants of the isolated domain [69] and in the full enzyme [57] showed that neither the folding of the GTPase domain nor the dimeric quaternary structure are largely altered by GTP addition. The secondary structure changes in the whole enzyme evidenced by CD spectroscopy were modest, showing that the overall folding does not change. Taken together, all these results suggest that GTP binding induces local conformational changes in the domain and changes in protein backbone dynamics that radiate to the active site. These subtle changes in conformation and backbone dynamics likely play a key role in the regulation of the interaction with the other maturases and/or hydrogenase. Intriguingly, it has been reported that the presence of GTP affects the EPR spectral properties of the HydF [4Fe-4S] cluster [68], suggesting a communication between the GTPase domain and the FeS cluster domain. Thus, another possibility is that the GTP-induced conformational switches could instead modulate the coordination and orientation of the [4Fe-4S] cluster, switching from the four-coordination state with the carboxylate bound to a three-coordination states ready to bind the [2Fe]_H_ precursors. Further work is needed to have more information on the actual mechanism.

## 3. The Overall Process

The function of the individual maturases has been the object of extended work, and in the last few years the knowledge of HydG and HydF proteins has significantly progressed. Quite lacking, on the contrary, is the knowledge of the interactions between the different maturases, the sequence of individual steps, and both the stoichiometry and the regulatory mechanisms of the overall process. Due to the multistep nature of the molecular pathway leading to the [FeFe]-hydrogenase maturation described above, a close and coordinate network of protein interactions between several partners must be achieved. The structural features and the dynamic behavior of HydF as scaffold and carrier assign to this protein a key role along the entire [FeFe]-hydrogenase maturation pathway and indicate its capability to establish functional interaction with all the players of this process. These binding events were at first inferred from co-purification of HydE and HydG with HydF [36] and then confirmed and quantified in vitro through a combination of Surface Plasmon Resonance and co-purification experiments using recombinant proteins from *C. acetobutylicum* [66]. The dissociation constants of HydE and HydG interacting with HydF have been determined both in the absence and in the presence of a non-hydrolysable GTP analogue. The study showed that HydE has a ten times higher affinity for HydF than HydG, both with and without GTP, and that HydG cannot interact with HydF if HydE is bound to it. This suggests that the interactions of HydE and HydG with the HydF scaffold are distinct events occurring in a precise functional order and would be fully consistent with the model proposed below. Nevertheless, the dissociation constants are relatively high, implying that the interaction of HydF with the other maturases is not very strong, as expected for a protein that acts as a scaffold for up to three other proteins (HydG, HydE, HydA). The role of GTP binding is less clear: while both proteins have slightly higher affinity for HydF when GTP is present, the difference is less than an order of magnitude. Together with the other assays used in the work, the authors suggested that HydE and HydG bind separately to HydF and not cooperatively, thereby ruling out the possibility of a ternary complex [66]. It is still unknown, however, if there is an interaction between HydG and HydE occurring before either interacts with HydF. 

**Figure 6:**
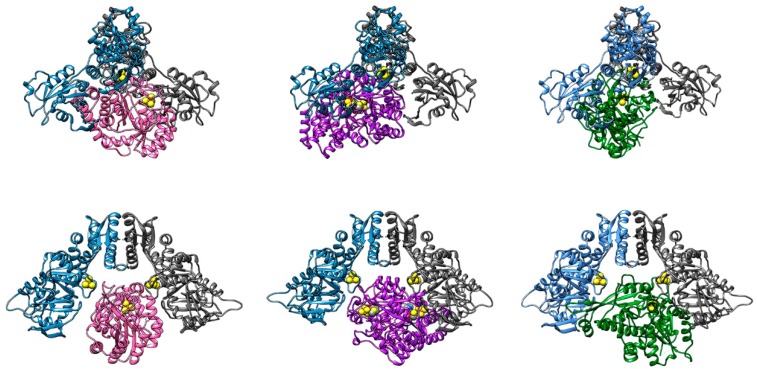
Molecular docking of reconstructed HydF from *T. melaniensis* (monomers in blue and grey) with: left, HydE (pink) from *T. maritima* (pdb.id 3CIW); center, HydG (purple) from *T. italicus* (pdb.id 4WCX); right, HydA (green) from *C. reinhardtii* (pdb.id 3LX4). Top and bottom show two views of the same docking pose rotated by 90°. All proteins show the active FeS clusters in sphere representation.

Since no direct evidence of the role of the cavity formed by the dimeric structure of HydF as the protein-protein interaction interface has been obtained so far, we used rigid-body molecular docking to test this hypothesis, which is relevant for the discussion of the overall maturation pathway. To perform the docking procedure, we docked together proteins from different organisms since there is no common organism for which all structures have been obtained. Note that performing the docking simulations of proteins from different organisms is meaningful since it has been shown that mixing maturases and HydA of different species still yields a functional hydrogenase [70]. For HydF, we used the reconstructed structure from *T. melaniensis* described above. Then, we chose the structures of the maturases for which the primary sequence had the highest homology with HydF from *T. melaniensis*: for HydE, the structure from *T. maritima* (pdb.id 3CIW) and for HydG, the structure from *T. italicus* (pdb.id 4WCX). For HydA, we chose the structure from *C. reinhardtii* (pdb.id 3LX4), since it has the smallest complete hydrogenase domain of the available structures. The soft rigid-body docking procedure, adapted from [71], was performed using the webservers of both PatchDock [72] (in tandem FireDock [73]) and ZDOCK [74]. The full results of these simulations will be further refined and presented in a future work, we here report the preliminary results of our analysis in Figure 6, where we chose three docking poses that show that indeed HydE, HydG, and HydA are all able to dock in the “cavity” formed by the dimer of HydF. However, these are rigid body docking simulations, dynamics are not accounted for, and some physiologically relevant docking poses, resulting from different conformations of flexible structural elements, might be lost. Additionally, we used the structure of HydF in the absence of GTP, and since the full structural changes induced by GTP binding are not known, the results might be very different in the GTP-bound state.

**Figure 7:**
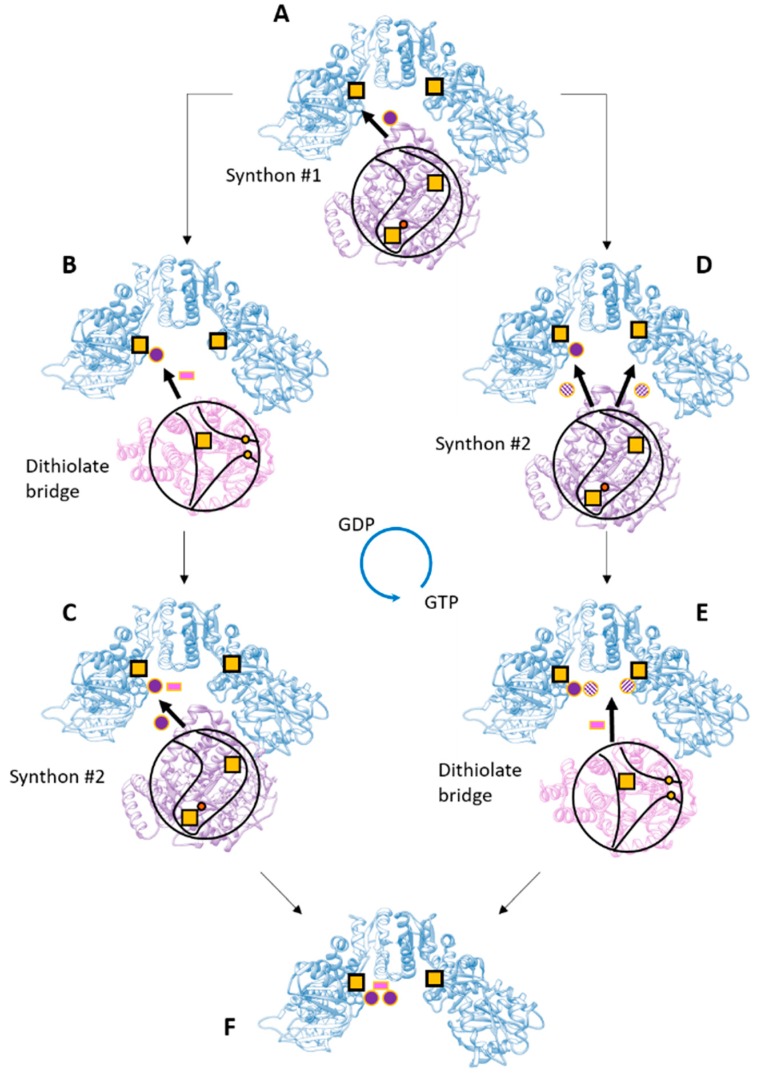
Possible sequence of events leading to the [2Fe]_H_ cluster assembly. In this hypothesis, HydG (purple) and HydE (pink) bind to HydF (blue) in separate steps. For the description of the steps please refer to the text. The cavities of HydE and HydG are shown schematically in black lines with the inner cavities outlined and the putative access points shown; yellow squares, [4Fe-4S] clusters; yellow dots, the cysteines of HydE; orange dot, the labile iron in HydG; purple dots, the synthon; pink rectangle, the dithiolate bridge.

Based on the evidence gathered so far, we propose two speculative models of the overall process that are schematically shown in Figure 7. These models provide for a central role of HydF that receives all parts of the cluster and assembles them, with separate binding events of HydG and HydE. As shown in Figure 1, the overall maturation of the [2Fe]_H_ cluster implies a stoichiometry for the maturases that depends on the protein: HydG needs four turnovers at the first active site and two at the second site to produce the two synthons necessary for the complete cluster, while only a single turnover is expected to be necessary for the formation of the dithiolate bridge by HydE. It seems unlikely that the reactive dithiolate bridge is delivered to HydF before a synthon is bound, therefore it is reasonable that the first step (Figure 7A) always involves the binding of HydG to HydF and the delivery of a synthon. The synthon could easily bind to the [4Fe-4S] cluster of HydF via the cyanide ligand like in the complete [2Fe]_F_ structure. It has to be noted that the only access to the inner cavity of HydG is the single channel on top of the cavity, where both substrate and products are thought to pass, and therefore the access to the active site of HydG would be sterically impossible when it is bound to HydF. Then, the synthesis and delivery of the second synthon imply a dissociation and re-association of HydG. Following the binding of the first synthon two possibilities exist: (1) HydE delivers the dithiolate bridge (Figure 7B) before a second HydG comes to deliver the second synthon to the partially formed [2Fe]_F_ cluster (Figure 7C); and (2) HydG binds to deliver the second synthon (Figure 7D) before HydE comes in to clip the two synthons together via the dithiolate bridge (Figure 7E). In the latter case, the second synthon could either be attached to the to the first one, implying a rearrangement of the ligands, or it could be delivered to the empty [4Fe-4S] cluster of the other HydF monomer and then coupled by HydE. The presence of two individual synthons in the two monomers of HydF is attractive, since it would provide another justification for its homo-dimeric structure besides the presence of the central cavity where the other maturases could dock. In both cases additional regulatory mechanisms, i.e., GTP binding, changes in protein backbone dynamics, and/or a conformational change of the cluster region following the binding of the first synthon, must modulate the binding affinity of the other maturases to HydF.

A further alternative mechanism would provide for HydG and HydE to interact first, assembling the whole [2Fe] sub-cluster possibly making use of the secondary cluster binding motif of HydE. Then, HydE would deliver the complete cluster to HydF. We performed docking simulations on a possible HydG-HydE complex, but the results do not show any preferential binding interface between the two proteins. However, this mechanism cannot be excluded, and experiments on HydE-HydG binding affinity would be needed to verify this hypothesis.

## Abbreviations

EPR	Electron Paramagnetic Resonance
GTP	Guanosine-5′-Triphosphate
GDP	Guanosine-5′-Diphosphate
SAM	S-adenosyl-L-Methionine
TIM	Triose-Phosphate Isomerase
DHG	Dehydroglycine
dAdoH	5′- Deoxyadenosine
4OB•	4-Oxidobenzyl Radical

## References

[1] McPherson I.J., Vincent K.A. (2014). Electrocatalysis by Hydrogenases: Lessons for Building Bio-Inspired Devices. J. Braz. Chem. Soc..

[2] Morra S., Valetti F., Gilardi G. (2017). [FeFe]-hydrogenases as biocatalysts in bio-hydrogen production. Rend. Lincei.

[3] Khan M.A., Ngo H.H., Guo W., Liu Y., Zhang X., Guo J., Chang S.W., Nguyen D.D., Wang J. (2018). Biohydrogen production from anaerobic digestion and its potential as renewable energy. Renew. Energy.

[4] Reeve H.A., Ash P.A., Park H., Huang A., Posidias M., Tomlinson C., Lenz O., Vincent K.A. (2017). Enzymes as modular catalysts for redox half-reactions in H_2_-powered chemical synthesis: From biology to technology. Biochem. J..

[5] Esmieu C., Raleiras P., Berggren G. (2017). From protein engineering to artificial enzymes—Biological and biomimetic approaches towards sustainable hydrogen production. Sustain. Energy Fuels.

[6] Greening C., Biswas A., Carere C.R., Jackson C.J., Taylor M.C., Stott M.B., Cook G.M., Morales S.E. (2016). Genomic and metagenomic surveys of hydrogenase distribution indicate H_2_ is a widely utilised energy source for microbial growth and survival. ISME J..

[7] Vignais P.M., Billoud B. (2007). Occurrence, classification, and biological function of hydrogenases: An overview. Chem. Rev..

[8] Lubitz W., Ogata H., Rüdiger O., Reijerse E. (2014). Hydrogenases. Chem. Rev..

[9] Corr M.J., Murphy J.A. (2011). Evolution in the understanding of [Fe]-hydrogenase. Chem. Soc. Rev..

[10] Ogata H., Lubitz W., Higuchi Y. (2016). Structure and function of [NiFe] hydrogenases. J. Biochem..

[11] Mulder D.W., Shepard E.M., Meuser J.E., Joshi N., King P.W., Posewitz M.C., Broderick J.B., Peters J.W. (2011). Insights into [FeFe]-hydrogenase structure, mechanism, and maturation. Structure.

[12] Baltazar C.S.A., Marques M.C., Soares C.M., DeLacey A.M., Pereira I.A.C., Matias P.M. (2011). Nickel-iron-selenium hydrogenases—An overview. Eur. J. Inorg. Chem..

[13] Vincent K.A., Parkin A., Armstrong F.A. (2007). Investigating and exploiting the electrocatalytic properties of hydrogenases. Chem. Rev..

[14] Marques M.C., Coelho R., De Lacey A.L., Pereira I.A.C., Matias P.M. (2010). The three-dimensional structure of [nifese] hydrogenase from desulfovibrio vulgaris hildenborough: A hydrogenase without a bridging ligand in the active site in its oxidised, “as-isolated” state. J. Mol. Biol..

[15] De Lacey A.L., Fernández V.M., Rousset M., Cammack R. (2007). Activation and inactivation of hydrogenase function and the catalytic cycle: Spectroelectrochemical studies. Chem. Rev..

[16] Goldet G., Brandmayr C., Stripp S.T., Happe T., Cavazza C., Fontecilla-Camps J.C., Armstrong F.A. (2009). Electrochemical kinetic investigations of the reactions of [FeFe]-hydrogenases with carbon monoxide and oxygen: Comparing the importance of gas tunnels and active-site electronic/redox effects. J. Am. Chem. Soc..

[17] Stripp S.T., Goldet G., Brandmayr C., Sanganas O., Vincent K.A., Haumann M., Armstrong F.A., Happe T. (2009). How oxygen attacks [FeFe] hydrogenases from photosynthetic organisms. Proc. Natl. Acad. Sci. USA.

[18] Lambertz C., Leidel N., Havelius K.G.V., Noth J., Chernev P., Winkler M., Happe T., Haumann M. (2011). O_2_ reactions at the six-iron active site (H-cluster) in [FeFe]-hydrogenase. J. Biol. Chem..

[19] Rodríguez-Maciá P., Reijerse E.J., Van Gastel M., Debeer S., Lubitz W., Rüdiger O., Birrell J.A. (2018). Sulfide Protects [FeFe] Hydrogenases from O_2_. J. Am. Chem. Soc..

[20] Oughli A.A., Vélez M., Birrell J.A., Schuhmann W., Lubitz W., Plumeré N., Rüdiger O. (2018). Viologen-modified electrodes for protection of hydrogenases from high potential inactivation while performing H2oxidation at low overpotential. Dalt. Trans..

[21] Caserta G., Papini C., Adamska-Venkatesh A., Pecqueur L., Sommer C., Reijerse E., Lubitz W., Gauquelin C., Meynial-Salles I., Pramanik D. (2018). Engineering an [FeFe]-Hydrogenase: Do Accessory Clusters Influence O_2_ Resistance and Catalytic Bias?. J. Am. Chem. Soc..

[22] Apfel U.P., Weigand W. (2013). Biomimetic assembly of the [FeFe] hydrogenase: Synthetic mimics in a biological shell. ChemBioChem.

[23] Artero V., Berggren G., Atta M., Caserta G., Roy S., Pecqueur L., Fontecave M. (2015). From Enzyme Maturation to Synthetic Chemistry: The Case of Hydrogenases. Acc. Chem. Res..

[24] Birrell J.A., Rüdiger O., Reijerse E.J., Lubitz W. (2017). Semisynthetic Hydrogenases Propel Biological Energy Research into a New Era. Joule.

[25] Stripp S.T., Happe T. (2009). How algae produce hydrogen—News from the photosynthetic hydrogenase. Dalt. Trans..

[26] Haumann M., Stripp S.T. (2018). The Molecular Proceedings of Biological Hydrogen Turnover. Acc. Chem. Res..

[27] Schilter D., Rauchfuss T.B. (2013). And the winner is...azadithiolate: An amine proton relay in the [FeFe] hydrogenases. Angew. Chem. Int. Ed..

[28] Adamska-Venkatesh A., Roy S., Siebel J.F., Simmons T.R., Fontecave M., Artero V., Reijerse E., Lubitz W. (2015). Spectroscopic Characterization of the Bridging Amine in the Active Site of [FeFe] Hydrogenase Using Isotopologues of the H.-Cluster. J. Am. Chem. Soc..

[29] Lill R. (2009). Function and biogenesis of iron-sulphur proteins. Nature.

[30] Posewitz M.C., King P.W., Smolinski S.L., Zhang L., Seibert M., Ghirardi M.L. (2004). Discovery of two novel radical S-adenosylmethionine proteins required for the assembly of an active [Fe] hydrogenase. J. Biol. Chem..

[31] Mulder D.W., Ortillo D.O., Gardenghi D.J., Naumov A.V., Ruebush S.S., Szilagyi R.K., Huynh B., Broderick J.B., Peters J.W. (2009). Activation of HydAΔEFG requires a preformed [4Fe-4S] cluster. Biochemistry.

[32] Nicolet Y., Fontecilla-Camps J.C. (2012). Structure-function relationships in [FeFe]-hydrogenase active site maturation. J. Biol. Chem..

[33] Peters J.W., Schut G.J., Boyd E.S., Mulder D.W., Shepard E.M., Broderick J.B., King P.W., Adams M.W.W. (2015). [FeFe]- and [NiFe]-hydrogenase diversity, mechanism, and maturation. Biochim. Biophys. Acta-Mol. Cell. Res..

[34] King P.W., Posewitz M.C., Ghirardi M.L., Seibert M., Al K.E.T., Acteriol J.B. (2006). Functional Studies of [FeFe] Hydrogenase Maturation in an Escherichia coli Biosynthetic System Functional Studies of [FeFe] Hydrogenase Maturation in an Escherichia coli Biosynthetic System. J. Bacteriol..

[35] Bourne H.R., Sanders D.A., McCormick F. (1991). The GTPase superfamily: Conserved structure and molecular mechanism. Nature.

[36] McGlynn S.E., Shepard E.M., Winslow M.A., Naumov A.V., Duschene K.S., Posewitz M.C., Broderick W.E., Broderick J.B., Peters J.W. (2008). HydF as a scaffold protein in [FeFe] hydrogenase H-cluster biosynthesis. FEBS Lett..

[37] Czech I., Silakov A., Lubitz W., Happe T. (2010). The [FeFe]-hydrogenase maturase HydF from Clostridium acetobutylicum contains a CO and CN-ligated iron cofactor. FEBS Lett..

[38] Betz J.N., Boswell N.W., Fugate C.J., Holliday G.L., Akiva E., Scott A.G., Babbitt P.C., Peters J.W., Shepard E.M., Broderick J.B. (2015). Hydrogenase maturation: Insights into the role hyde plays in dithiomethylamine biosynthesis. Biochemistry.

[39] Nicolet Y., Rubach J.K., Posewitz M.C., Amara P., Mathevon C., Atta M., Fontecave M., Fontecilla-Camps J.C. (2008). X-ray structure of the [FeFe]-hydrogenase maturase HydE from Thermotoga maritima. J. Biol. Chem..

[40] Nicolet Y., Rohac R., Martin L., Fontecilla-Camps J.C. (2013). X-ray snapshots of possible intermediates in the time course of synthesis and degradation of protein-bound Fe4S4 clusters. Proc. Natl. Acad. Sci. USA.

[41] Rao G., Tao L., Suess D.L.M., Britt R.D. (2018). A [4Fe–4S]-Fe(CO)(CN)-l-cysteine intermediate is the first organometallic precursor in [FeFe] hydrogenase H-cluster bioassembly. Nat. Chem..

[42] Nicolet Y., Pagnier A., Zeppieri L., Martin L., Amara P., Fontecilla-Camps J.C. (2015). Crystal structure of HydG from carboxydothermus hydrogenoformans: A trifunctional [FeFe]-Hydrogenase maturase. Chem. Biol. Chem..

[43] Dinis P., Suess D.L.M., Fox S.J., Harmer J.E., Driesener R.C., De La Paz L., Swartz J.R., Essex J.W., Britt R.D., Roach P.L. (2015). X-ray crystallographic and EPR spectroscopic analysis of HydG, a maturase in [FeFe]-hydrogenase H.-cluster assembly. Proc. Natl. Acad. Sci. USA.

[44] Kuchenreuther J.M., Myers W.K., Stich T.A., George S.J., Nejatyjahromy Y., Swartz J.R., Britt R.D. (2013). A Radical Intermediate in Tyrosine of FeFe Hydrogenase. Science.

[45] Kuchenreuther J.M. (2014). The HydG Enzyme Generates an. Science.

[46] Suess D.L.M., Kuchenreuther J.M., De La Paz L., Swartz J.R., Britt R.D. (2016). Biosynthesis of the [FeFe] Hydrogenase H Cluster: A Central Role for the Radical SAM Enzyme HydG. Inorg. Chem..

[47] Suess D.L.M., Pham C.C., Bürstel I., Swartz J.R., Cramer S.P., Britt R.D. (2016). The Radical SAM Enzyme HydG Requires Cysteine and a Dangler Iron for Generating an Organometallic Precursor to the [FeFe]-Hydrogenase, H.-Cluster. J. Am. Chem. Soc..

[48] Cendron L., Berto P., D’Adamo S., Vallese F., Govoni C., Posewitz M.C., Giacometti G.M., Costantini P., Zanotti G. (2011). Crystal structure of HydF scaffold protein provides insights into [FeFe]-hydrogenase maturation. J. Biol. Chem..

[49] Shepard E.M., Duffus B.R., George S.J., Mcglynn S.E., Challand M.R., Swanson K.D., Roach P.L., Cramer S.P., Peters J.W., Broderick J.B. (2010). [FeFe]-Hydrogenase Maturation: HydG-Catalyzed Synthesis of Carbon Monoxide. J. Am. Chem. Soc..

[50] Brazzolotto X., Rubach J.K., Gaillard J., Gambarelli S., Atta M., Fontecave M. (2006). The [Fe-Fe]-hydrogenase maturation protein HydF from Thermotoga maritima is a GTPase with an iron-sulfur cluster. J. Biol. Chem..

[51] Czech I., Stripp S., Sanganas O., Leidel N., Happe T., Haumann M. (2011). The [FeFe]-hydrogenase maturation protein HydF contains a H-cluster like [4Fe4S]-2Fe site. FEBS Lett..

[52] Berto P., Di Valentin M., Cendron L., Vallese F., Albertini M., Salvadori E., Giacometti G.M., Carbonera D., Costantini P. (2012). The [4Fe-4S]-cluster coordination of [FeFe]-hydrogenase maturation protein HydF as revealed by EPR and HYSCORE spectroscopies. Biochim. Biophys. Acta-Bioenergy.

[53] Berggren G., Garcia-Serres R., Brazzolotto X., Clemancey M., Gambarelli S., Atta M., Latour J.M., Hernández H.L., Subramanian S., Johnson M.K. (2014). An EPR/HYSCORE, Mössbauer, and resonance Raman study of the hydrogenase maturation enzyme HydF: A model for N-coordination to [4Fe-4S] clusters. J. Biol. Inorg. Chem..

[54] Albertini M., Vallese F., Di Valentin M., Berto P., Giacometti G.M., Costantini P., Carbonera D. (2014). The proton iron-sulfur cluster environment of the [FeFe]-hydrogenase maturation protein HydF from Thermotoga neapolitana. Int. J. Hydrogen Energy.

[55] Albertini M., Galazzo L., Maso L., Vallese F., Berto P., De Rosa E., Di Valentin M., Costantini P., Carbonera D. (2015). Characterization of the [FeFe]-Hydrogenase Maturation Protein HydF by EPR Techniques: Insights into the Catalytic Mechanism. Top. Catal..

[56] Caserta G., Pecqueur L., Adamska-Venkatesh A., Papini C., Roy S., Artero V., Atta M., Reijerse E., Lubitz W., Fontecave M. (2017). Structural and functional characterization of the hydrogenase-maturation HydF protein. Nat. Chem. Biol..

[57] Galazzo L., Maso L., De Rosa E., Bortolus M., Doni D., Acquasaliente L., Filippis V.D.V.D., Costantini P., Carbonera D. (2017). Identifying conformational changes with site-directed spin labeling reveals that the GTPase domain of HydF is a molecular switch. Sci. Rep..

[58] Ash M.R., Maher M.J., Mitchell Guss J., Jormakka M. (2012). The cation-dependent G-proteins: In a class of their own. FEBS Lett..

[59] Pettersen E.F., Goddard T.D., Huang C.C., Couch G.S., Greenblatt D.M., Meng E.C., Ferrin T.E. (2004). UCSF Chimera - A visualization system for exploratory research and analysis. J. Comput. Chem..

[60] Ash M.R., Guilfoyle A., Clarke R.J., Guss J.M., Maher M.J., Jormakka M. (2010). Potassium-activated GTPase reaction in the G protein-coupled ferrous iron transporter B. J. Biol. Chem..

[61] Broderick J.B., Byer A.S., Duschene K.S., Duffus B.R., Betz J.N., Shepard E.M., Peters J.W. (2014). H-Cluster assembly during maturation of the [FeFe]-hydrogenase. J. Biol. Inorg. Chem..

[62] Shepard E.M., Byer A.S., Aggarwal P., Betz J.N., Scott A.G., Shisler K.A., Usselman R.J., Eaton G.R., Eaton S.S., Broderick J.B. (2017). Electron Spin Relaxation and Biochemical Characterization of the Hydrogenase Maturase HydF: Insights into [2Fe-2S] and [4Fe-4S] Cluster Communication and Hydrogenase Activation. Biochemistry.

[63] Shepard E.M., Byer A.S., Broderick J.B. (2017). Iron-Sulfur Cluster States of the Hydrogenase Maturase HydF. Biochemistry.

[64] Shepard E.M., Byer A.S., Betz J.N., Peters J.W., Broderick J.B. (2016). A redox active [2Fe-2S] cluster on the hydrogenase maturase HydF. Biochemistry.

[65] Scott A.G., Szilagyi R.K., Mulder D.W., Ratzlo W., Byer A.S., King P.W., Broderick W.E., Shepard M., Broderick J.B. (2018). Compositional and structural insights into the nature of the H-cluster precursor on HydF. Dalt. Trans..

[66] Vallese F., Berto P., Ruzzene M., Cendron L., Sarno S., De Rosa E., Giacometti G.M., Costantini P. (2012). Biochemical analysis of the interactions between the proteins involved in the [FeFe]-hydrogenase maturation process. J. Biol. Chem..

[67] Leipe D.D., Wolf Y.I., Koonin E.V., Aravind L. (2002). Classification and evolution of P-loop GTPases and related ATPases. J. Mol. Biol..

[68] Shepard E.M., McGlynn S.E., Bueling A.L., Grady-Smith C.S., George S.J., Winslow M.A., Cramer S.P., Peters J.W., Broderick J.B. (2010). Synthesis of the 2Fe subcluster of the [FeFe]-hydrogenase H cluster on the HydF scaffold. Proc. Natl. Acad. Sci. USA.

[69] Maso L., Galazzo L., Vallese F., Di Valentin M., Albertini M., De Rosa E., Giacometti G.M., Costantini P., Carbonera D. (2015). A conformational study of the GTPase domain of [FeFe]-hydrogenase maturation protein HydF by PELDOR spectroscopy. Appl. Magn. Reson..

[70] McGlynn S.E., Ruebush S.S., Naumov A., Nagy L.E., Dubini A., King P.W., Broderick J.B., Posewitz M.C., Peters J.W. (2007). In vitro activation of [FeFe] hydrogenase: New insights into hydrogenase maturation. J. Biol. Inorg. Chem..

[71] De Toni L., Guidolin D., De Filippis V., Tescari S., Strapazzon G., Rocca M.S., Ferlin A., Plebani M., Foresta C. (2016). Osteocalcin and sex hormone binding globulin compete on a specific binding site of GPRC6A. Endocrinology.

[72] Schneidman-Duhovny D., Inbar Y., Nussinov R., Wolfson H.J. (2005). PatchDock and SymmDock: Servers for rigid and symmetric docking. Nucleic Acids Res..

[73] Andrusier N., Nussinov R., Wolfson H.J. (2007). FireDock: Fast interaction refinement in molecular docking. Proteins Struct. Funct. Genet..

[74] Pierce B.G., Wiehe K., Hwang H., Kim B.H., Vreven T., Weng Z. (2014). ZDOCK server: Interactive docking prediction of protein-protein complexes and symmetric multimers. Bioinformatics.

